# Altered Transcriptome Signature in Primary Human Myotubes Exposed to Inclusion Body Myositis Serum: A Pilot Case Comparison of Anti-cN1A Positive and Negative Sera

**DOI:** 10.3390/muscles4040053

**Published:** 2025-11-10

**Authors:** Nataliya Slater, Abha Chopra, Ramesh Ram, Abbie Adams, Frank L. Mastaglia, Merrilee Needham, Jerome D. Coudert

**Affiliations:** 1 Personalised Medicine Center, School of Medical, Molecular and Forensic Sciences, Murdoch University, Murdoch, WA 6150, Australia; abha.chopra@murdoch.edu.au (A.C.); ramesh.ram@murdoch.edu.au (R.R.); merrilee.needham@health.wa.gov.au (M.N.); jerome.coudert@utoulouse.fr (J.D.C.); 2 Perron Institute for Neurological and Translational Science, Nedlands, WA 6009, Australia; 3 Centre for Neuromuscular & Neurological Disorders, University of Western Australia, Crawley, WA 6009, Australia; 4 School of Medicine, University of Notre Dame Australia, Fremantle, WA 6160, Australia; 5 Department of Neurology, Fiona Stanley Hospital, Murdoch, WA 6150, Australia

**Keywords:** inclusionbody myositis, cytosolic 5′-nucleotidase 1A, NT5C1A, anti-cN1A, autoantibodies, primary human myotubes, inflammatory myopathy

## Abstract

Inclusion body myositis (IBM) is a late-onset, treatment-resistant inflammatory myopathy. Approximately half of IBM patients develop autoantibodies against cytosolic $5^{\prime }$-nucleotidase 1A (cN1A), but their role in disease pathogenesis remains unclear. This pilot study examined the effects of anti-cN1A-positive IBM serum on human primary myotubes’ transcriptome profile, using anti-cN1A-negative IBM and healthy sera as controls. Exposure to anti-cN1A-positive serum altered the expression of 1126 genes, with upregulation of adaptive immune response genes, notably *CTSH* and *CTSZ*, encoding cathepsins H and Z. These findings were validated using a publicly available independent dataset comprising transcriptomes from fresh muscle tissue samples. *NT5C1A* mRNA, which encodes cN1A, was not detected in cultured myotubes regardless of the presence of autoantibodies. The findings suggest distinct pathological mechanisms in anti-cN1A-positive IBM, independent of direct antibody-target interactions. The role of cathepsins in IBM pathogenesis warrants further investigation.

## 1. Introduction

Inclusion body myositis (IBM) is a rare acquired inflammatory myopathy occurring in older adults, with a typical presentation after the age of 45 [1]. The disease is characterised by progressive tissue wastage and weakness of selective muscle groups. There are currently no effective therapies or reliable biomarkers for IBM, highlighting a substantial unmet clinical need. While the precise disease aetiology remains unclear, accumulating evidence suggests a complex interplay between immune-mediated inflammation and degeneration associated with impaired proteostasis and mitochondrial dysfunction [2]. One of the immunological features of IBM is the presence of autoantibodies targeting cytosolic $5^{\prime }$-nucleotidase 1A (cN1A), reported in 33–80% of patients [3,4,5,6,7,8]. Although anti-cN1A antibodies have been incorporated into the most recent ENMC diagnostic criteria for IBM [1], they are not considered a disease biomarker due to their limited specificity [9]. These antibodies have also been detected in up to 36% of patients with other conditions and in approximately 5% of the healthy population [6].

Cytosolic $5^{\prime }$-nucleotidase 1A (cN1A), the target of autoantibodies in IBM, belongs to the $5^{\prime }$-nucleotidase enzyme family, which plays a critical role in the nucleotide salvage pathway. These enzymes recycle nucleosides produced during the degradation of RNA and DNA, enabling their reuse in nucleotide synthesis and DNA repair [10]. cN1A is highly expressed in skeletal and cardiac muscle, where it primarily acts on adenosine monophosphate (AMP) as its substrate [11,12,13].

Evidence for the role of anti-cN1A antibodies in IBM pathogenesis and their functional consequences on skeletal muscle remains limited. Tawara and colleagues investigated the pathogenic effects of anti-cN1A IgG in a rhabdomyosarcoma cell line [14]. In their study, human rhabdomyosarcoma cells were co-cultured with IgG from anti-cN1A-positive and -negative IBM patients. The autoantibody effects were examined at the protein level, revealing a significant reduction in intracellular cN1A. Additionally, levels of the autophagy-related protein p62 (also known as EBIAP, ZIP3, and SQSTM1/Sequestosome-1), whose aggregation is a pathological hallmark of IBM [15], were elevated in antibody-treated samples [14]. These findings raise intriguing questions about whether anti-cN1A IgG passively crosses intact cell membranes or is actively transported into cells, as well as whether its interaction with intracellular cN1A leads to functional neutralisation or degradation through yet undefined mechanisms. Nevertheless, the tumorigenic origin of rhabdomyosarcoma cells necessitates careful interpretation when extrapolating these results to normal or diseased skeletal muscle physiology.

Myotubes differentiated from human primary myoblasts may provide a more physiologically relevant in vitro model for investigating muscle-specific responses to disease-associated factors. In the context of IBM, exposure of myotube cultures to patient-derived serum offers a controlled system to explore potential pathogenic mechanisms mediated by circulating autoantibodies and other immune mediators. This study aimed to provide a proof-of-concept for the suitability of the two-dimensional (2D) primary myotube culture model for investigating the effects of anti-cN1A-positive IBM serum on skeletal muscle gene expression profile. Through transcriptomic analysis, we seek to identify differentially expressed genes and pathways that might contribute to muscle dysfunction in IBM, particularly in the context of antibody-positive patients. Understanding these molecular alterations could provide further insights into the disease pathogenesis and identify potential therapeutic targets.

## 2. Materials and Methods

### 2.1. Patients’ Informed Consent

Written informed consent was obtained from all participants prior to their inclusion in this study. The study was conducted in accordance with the ethical principles outlined in the Declaration of Helsinki. Participants were informed about the purpose of the study, the procedures involved, potential risks and benefits, and their right to withdraw at any time without consequence.

### 2.2. Serum Donor Characteristic

IBM patients were diagnosed with definite IBM by an experienced neurologist (M.N.) according to 2013 ENMC diagnostic criteria [16]. Anti-cN1A antibody serostatus was assessed using an in-house ELISA with full-length recombinant cN1A as antigen [17]. Both IBM patients were tested annually for at least three years, consistently maintaining their respective seropositive or seronegative status. We do not routinely test healthy control serum for anti-cN1A.

Demographic and clinical characteristics of the serum donors are presented in Table 1. All donors were male, selected to minimise variability related to circulating sex hormones.

### 2.3. Blood Collection and Serum Isolation

Sera was isolated from venous blood collected into serum separator tubes (BD Life Sciences, Australia). The tubes were centrifuged at 1500× *g* for 10 min at room temperature. Following centrifugation, the serum layer was carefully aspirated using a sterile transfer pipette and transferred into pre-labelled 1.5 mL microcentrifuge tubes (Merck, Darmstadt, Germany). Aliquots were stored at −80 °C.

### 2.4. Myoblasts Isolation and Expansion

**Figure 1:**
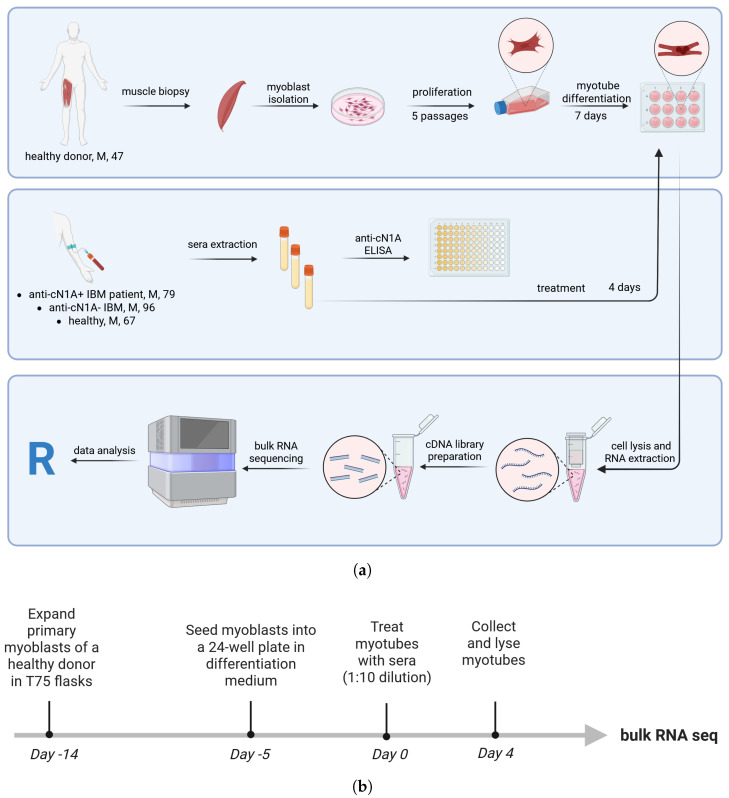
Graphical summary of the experimental design. (**a**) Experimental setup. The letter “M” following the donor description indicates male gender, and the subsequent number denotes donor age. (**b**) Timeline of the experiment. Figures were created with BioRender.

Experimental design is summarised in Figure 1. Primary human myoblasts were isolated from a biopsied Vastus medialis muscle of a healthy 46-year-old male following the established protocol [18]. Myoblasts were further expanded over several passages in vented T75 culture flasks (Thermo Fisher Scientific, Paisley, UK) coated with Matrigel (Corning Inc., Corning, NY, USA).

For coating, 4 mL of Matrigel was transferred into a flask and distributed over the entire surface. The flask was incubated at 37 °C for 1 h. After incubation, the excess matrix was collected, and the flask was immediately seeded with cells. Myoblasts were grown in expansion medium consisting of Ham’s F-10 Nutrient Mix (Gibco, Thermo Fisher Scientific, Waltham, MA, USA), 20% fetal calf serum (FCS, Serana Pty Ltd., Bunbury, WA, Australia), 0.5% chick embryo extract (US Biological Life Sciences, Salem, MA, USA), and 1% Penicillin-Streptomycin-L-Glutamine (Gibco, Thermo Fisher Scientific, Waltham, MA, USA). Cells were cultured to 70–80% confluency before passaging.

To passage adherent myoblasts, the medium was removed, and cells were washed with warm Phosphate-Buffered Saline (PBS, Gibco, Thermo Fisher Scientific, Waltham, MA, USA). Cells were then covered with 4 mL of warm 0.5% Trypsin-EDTA (Gibco, Thermo Fisher Scientific, Waltham, MA, USA) and incubated at 37 °C for 3–5 min. To detach the cells, the flask was gently tapped against a flat hand. Once most cells were rounded and floating, the trypsin solution was inactivated with 6 mL of high-glucose DMEM (Gibco, Thermo Fisher Scientific, Waltham, MA, USA) containing 10% horse serum (Gibco, Thermo Fisher Scientific, Auckland, New Zealand) and collected into a 15 mL conical tube. Cells were centrifuged at 2000× g for 10 min.

After resuspension of the cell pellet in a suitable volume of expansion medium to achieve 1–2 $\times 10^{6}$ myocytes per mL, cells were counted manually in a Neubauer chamber using the trypan blue exclusion method.

### 2.5. Myoblast Differentiation

After the fifth passage, myoblasts were seeded into a 24-well culture plate for differentiation. First, the wells were coated with 50 µg/mL of Poly-D- Lysine (Gibco, Thermo Fisher Scientific, USA) in PBS at 23 °C for 1 h. Then, a coating layer of 200 µL of Matrigel was applied at 37 °C for 1 h. Myoblasts were prepared in differentiation medium consisting of DMEM, 5% horse serum, and 1% Penicillin-Streptomycin-L-Glutamine, and seeded at 30,000 cells in 1 mL per well. Myoblasts were differentiated into multinuclear myotubes at 37 °C, 5% CO_2_ for seven days—a time sufficient to achieve termination of replication and stabilisation of gene expression [19].

### 2.6. Myotube Treatment

Once mature myotubes had formed, as confirmed by multinuclear bifurcated cell morphology, the medium was aspirated and replaced by differentiation medium (DMEM, 5% horse serum, 1% Penicillin-Streptomycin-L-Glutamine) supplemented with 10% *v/v* human sera derived from a healthy donor (HC), a seronegative IBM patient (IBM_Neg), and a seropositive IBM patient (IBM_Pos). The myotubes were treated with each serum in triplicate wells.

Untreated (UT) control samples were cultured in differentiation medium without human serum addition. The treatment conditions were distributed randomly across the plate to avoid batching effects due to uneven heat distribution or higher media evaporation in the outer perimeter wells. Treated myotubes were cultured at 37 °C, 5% CO_2_ for four days.

### 2.7. Cell Collection and Lysis

After four days, the medium was aspirated, and wells were washed with 1 mL warm PBS. To detach the cells, wells were treated with 200 µL of 0.5% Trypsin/EDTA for 3 min. The reaction was stopped by adding 300 µL of 10% horse serum/DMEM. Cells treated with the same serum (triplicate repeats) were then pooled into 15 mL conical tubes and pelleted as before. As much of the medium as possible was carefully removed with a pipette, and the cell pellet was lysed in 10 µL of mild lysis buffer containing 0.2% Triton X-100 (Sigma-Aldrich, St. Louis, MO, USA) and a recombinant ribonuclease inhibitor (Invitrogen, Thermo Fisher Scientific, Waltham, MA, USA), and immediately frozen at –80 °C until sequencing.

### 2.8. RNA Sequencing

Sequencing was performed by the Institute for Immunology and Infectious Diseases Medical Genomics Core Laboratory (Murdoch University, Perth, WA, Australia), a medical genomics facility accredited by the American Society for Histocompatibility and Immunogenetics (ASHI) and the Australian National Association of Testing Authorities (NATA).

RNA was extracted from cell lysates using Direct-zol RNA extraction kit (Zymo Research, Irvine, CA, USA) and subsequently depleted of ribosomal RNA using NEBNext rRNA depletion kit v2 (New England Biolabs, Ipswich, QLD, Australia).

Complementary DNA (cDNA) was synthesised from the ribosomal RNA-depleted RNA using an adapted SmartSeq assay, optimised from Picelli et al. [20]. This method involves incorporating a transfer switching oligo (TSO) adaptor site at the $3^{\prime }$ end of the RNA template during first-strand synthesis. Concurrently, an oligo-dT primer was used to target the polyadenylated (poly-A) tail at the $3^{\prime }$ end. Double-stranded cDNA was then generated using primers specific to both the $3^{\prime }$ poly-A tail and the $5^{\prime }$ adaptor sequence.

The resulting double-stranded cDNA was utilised for sequencing library preparation. Sequencing was conducted on the NovaSeq 6000 platform (Illumina Inc., San Diego, CA, USA) using 150 base pair paired-end chemistry. On average, 85 million reads were obtained per sample.

Quality-filtered reads were aligned to the RefSeq human reference genome HG38.97 using the CLCbio Genomics Workbench version 21 (Qiagen, Hilden, Germany). Gene-specific read counts were calculated using the featureCounts function in the RSubread R package version 2.22.1, employing Ensembl gene annotations corresponding to the HG38.97 reference genome. The resulting count matrix was processed to assign gene names as gene symbols.

### 2.9. Reads Pre-Processing

Gene counts were analysed using edgeR version 4.0.16 package in R version 4.3.3.

The following pre-processing steps were performed sequentially:

- the raw count matrix was transformed to $\log_{2}$ counts per million (CPM);
- lowly expressed genes were filtered using the filterByExpr function;
- the filtered data were normalised using the Trimmed Mean of M-values (TMM) method, which accounts for compositional biases arising from differences in library sizes, sequencing depth, or gene expression distributions across samples.
- genes were annotated using Bioconductor org.Hs.eg.db package v. 3.20.0 as protein-coding, long non-coding RNA or pseudogenes. The count matrix was subsequently filtered to retain only protein-coding genes.

### 2.10. Differentially Expressed Gene Analysis

To define the differentially expressed genes associated with anti-cN1A-positive serum treatment of primary human myotubes, the transcriptome under this condition was compared to each of the two control groups (anti-cN1A- negative and healthy serum treatments) using the EdgeR *exactTest* function. The untreated control group was excluded from the analysis, as the addition of human serum to the culture medium was found to significantly affect the expression of numerous genes relative to horse serum-containing medium. Consequently, only samples cultured with both horse and human sera were included in the analysis.

The Benjamini-Hochberg correction was used to adjust for multiple comparisons. Genes were considered differentially expressed if they were below the false-discovery rate (FDR) threshold of 0.05. Genes at the intersection of all comparisons represented differentially expressed genes of interest. Gene set enrichment analysis was conducted using Protein Analysis THrough Evolutionary Relationships (PANTHER) version 19.0 [21]. Unordered list of upregulated genes was interrogated against GO biological process complete. Functional classification of genes was also conducted.

## 3. Results

### 3.1. Myotubes Differentiation In Vitro

**Figure 2:**
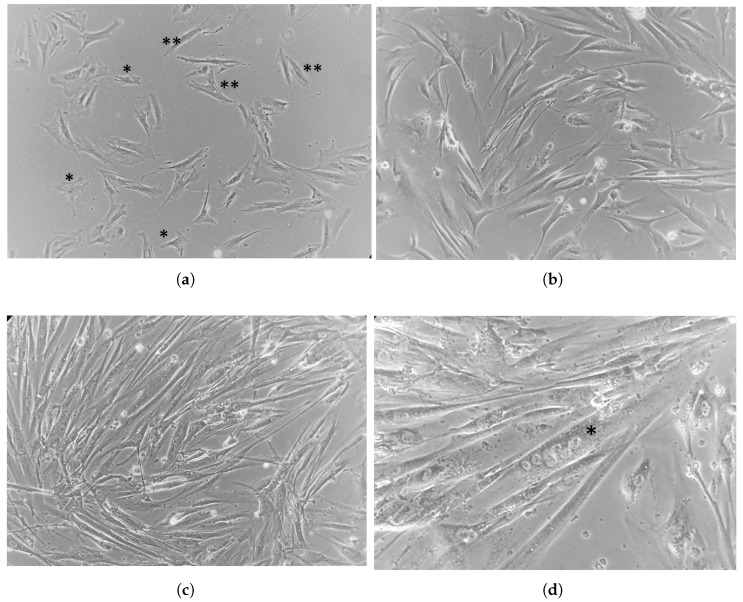
Representative brightfield images depicting the stages of primary human myoblast differentiation into myotubes. Images were captured using an Echo Revolve microscope: (**a**) Satellite cells (*) and elongated myoblasts (**), 10×. (**b**) myoblasts developing long projections, 10×. (**c**) fused myotubes in parallel orientation, 10×. (**d**) magnified image of a multinuclear myotube (*), 20×.

Isolated progenitor myocytes comprised satellite cells and elongated myoblasts (Figure 2a). In the early stages of differentiation, myoblasts continued to elongate and develop long projections (Figure 2b). Mature myotubes appeared as long, sometimes bifurcated cells oriented parallel to one another (Figure 2c). Post-fusion, these cells became long, thick, and contained multiple central nuclei (Figure 2c). Cells were identified as myoblasts or myotubes based on morphological characteristics and skeletal muscle specific gene expression (Table 2).

### 3.2. Differential Gene Expression

The total number of analysed genes after pre-processing was 12,323.

**Figure 3:**
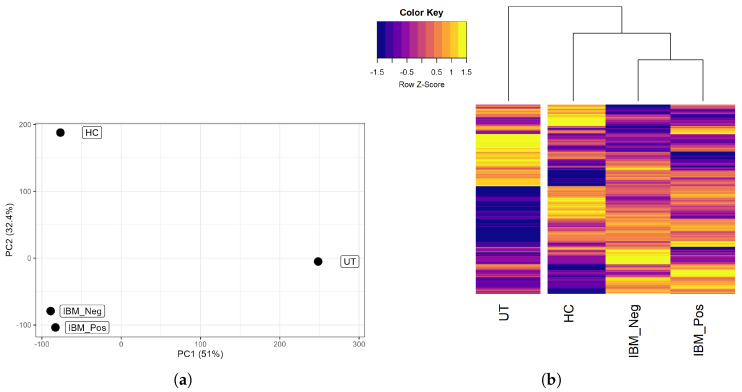
Gene expression in cultured myotubes treated with IBM (IBM_Neg or IBM_Pos) or healthy (HC) human serum or under baseline conditions (UT): (**a**) Principal component analysis of cultured myotube transcriptomes. (**b**) Hierarchical clustering of all expressed genes. Rows were clustered by Pearson correlation, columns were clustered by Spearman correlation.

The principal component analysis demonstrated a clear separation of the samples based on the top two principal components (Figure 3a). As expected, the two IBM samples clustered close to each other and away from the control samples.

Hierarchical clustering of protein-coding gene expression across all conditions tested revealed strikingly distinct expression patterns (Figure 3b). Notably, myotubes supplemented with human serum showed marked differences compared to the untreated controls. However, this variation is likely due to growth factors and other biologically active molecules in the serum, rather than the effects of anti-cN1A antibodies. Therefore, the differential gene expression analysis was restricted to samples supplemented with human serum excluding the untreated controls.

As samples cultured under identical conditions were pooled for sequencing, replicate gene counts were not available to estimate inter- and intra-sample dispersion. To address this limitation, the dispersion was calculated using a set of 21 housekeeping genes shared across the three comparison groups, as summarised in Supplementary Table S1. The dispersion value was 0.17, which is close to an expected value of 0.1 for genetically identical organisms such as the primary myotubes derived from a single donor used in this study.

**Figure 4:**
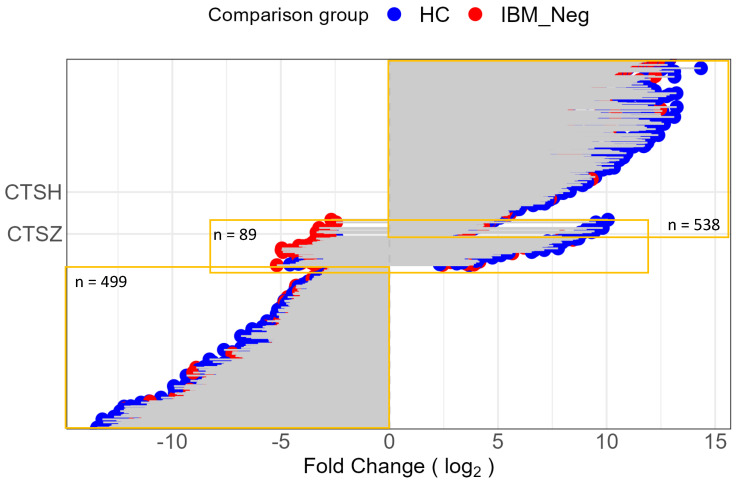
Differentially expressed genes (DEGs) in primary myotubes treated with anti-cN1A–positive serum (IBM_Pos), compared with anti-cN1A–negative serum (IBM_Neg) and healthy control serum (HC). Colours indicate the comparison group: blue = DEGs relative to HC; red = DEGs relative to IBM_Neg. Orange boxes highlight gene sets showing either concordant (up- or downregulated in both comparisons) or discordant (opposite direction) changes. Numbers denote the count of genes within each group.

Transcriptome analysis identified 3580 differentially expressed genes (DEGs) between myotubes treated with anti-cN1A-positive IBM serum and those treated with healthy serum, and 2227 DEGs between myocytes treated with anti-cN1A-positive and anti-cN1A-negative IBM sera. A total of 1126 DEGs were common to both comparisons (Figure 4).

Among the genes whose expression changed upon the treatment with anti-cN1A-positive serum when compared with both anti-cN1A-negative IBM serum and healthy serum treatments, 538 were upregulated, 498 were downregulated and, interestingly, 89 were changed in the opposite directions, for example upregulated when compared with healthy serum treatment and downregulated when compared with anti-cN1A negative serum or visa versa (Figure 4).

### 3.3. Upregulation of the Adaptive Immune Genes

The majority of the upregulated genes encoded metabolite interconversion enzymes (11.3%) and transcriptional regulators (11.2%) (Supplementary Figure S1). Among these DEGs, we identified two cysteine proteases, CTSH and CTSZ, that play roles in intracellular protein degradation, promoting antigen processing and initiation of adaptive immune responses. Treatment with IBM_Pos serum led to a marked increase in *CTSH* expression, approximately 222-fold higher than HC serum treatment and 7.5-fold higher than IBM_Neg serum treatment. *CTSZ* expression was elevated to a more moderate degree in both comparisons, increasing roughly 8-fold relative to each group.

**Figure 5:**
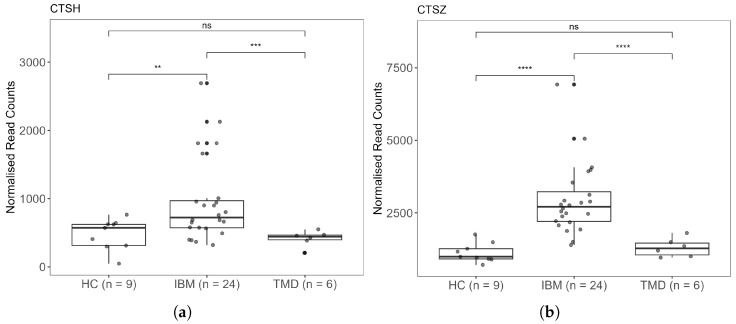
Comparison of *CTSH* (**a**) and *CTSZ* (**b**) expression in skeletal muscle biopsies from healthy donors (HC), patients with inclusion body myositis (IBM), and patients with tibial muscular dystrophy (TMD). RNA sequencing data were retrieved from GSE151757 (https://www.ncbi.nlm.nih.gov/geo/query/acc.cgi?acc=GSE151757). Pairwise comparisons were performed using the Student’s *t* test. Significance levels are indicated as follows: ns, *p* > 0.05; ** *p* ≤ 0.01; *** *p* ≤ 0.001; **** *p* ≤ 0.0001.

To assess the relevance of these findings to disease pathogenesis, we interrogated an RNA sequencing dataset generated by Johari and colleagues [22]. In this study, RNA was sequenced from muscle biopsies obtained from 24 IBM patients, six tibial muscular dystrophy (TMD) patients, and nine healthy controls (HC) without known muscle disease who underwent limb amputation (GSE151757 (https://www.ncbi.nlm.nih.gov/geo/query/acc.cgi?acc=GSE151757)). Consistent with our observations, analysis of this dataset demonstrated significant upregulation of *CTSH* and *CTSZ* in IBM samples compared with control groups (Figure 5).

### 3.4. Expression of Enzymes Within the Adenosine Salvage Pathway

**Figure 6:**
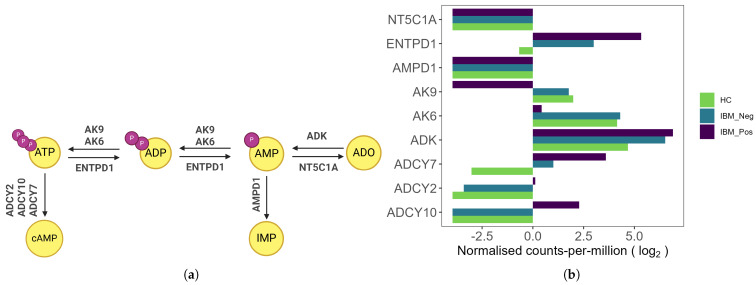
Enzymes involved in the adenosine salvage pathway. (**a**) Schematic illustrating the interconversion of adenosine triphosphate (ATP), diphosphate (ADP), monophosphate (AMP) and adenosine (ADO). ADCY—Adenylate Cyclase, AK—Adenylate Kinase, ENTPD—Ectonucleoside Triphosphate Diphosphohydrolase, AMPD—Adenosine Monophosphate Deaminase, ADK—Adenosine Kinase, NT5C1A—Cytosolic $5^{\prime }$-Nucleotidase 1A, cAMP—cyclic monophosphate, IMP—inosine monophosphate. (**b**) Normalised expression levels of key enzymes involved in the pathway under the tested conditions.

Cytosolic $5^{\prime }$-nucleotidase 1A (*NT5C1A*), the target of anti-cN1A antibodies in IBM, is an enzyme involved in the adenosine salvage pathway, illustrated in Figure 6a. This pathway consists of a series of enzymatic reactions that convert nucleoside phosphate precursors into the purine nucleoside adenosine (Ado). Figure 6b demonstrates the expression profiles of genes encoding enzymes participating in these interconversion reactions. Notably, the transcripts for *NT5C1A* and adenosine monophosphate deaminase (*AMPD1*), the two enzymes responsible for adenosine monophosphate (AMP) catabolism, were at undetectable levels in our RNA sequencing dataset across all three experimental conditions. Furthermore, adenylate kinases AK6 and AK9, which mediate the interconversion of adenosine diphosphate (ADP) and AMP, were significantly downregulated in the seropositive treatment group compared to the other groups. In contrast, transcripts of the three adenylate cyclases, ADCY2, ADCY7, and ADCY10, which catalyse the synthesis of cyclic adenosine monophosphate (cAMP), were significantly upregulated in the anti-cN1A-treated group relative to the other treatment conditions.

### 3.5. NT5C1A Expression Is Downregulated in Cultured Myotubes

To investigate whether the downregulation of *NT5C1A* was due to the culture process, we interrogated publicly available datasets comparing gene expression of skeletal muscle biopsies and cultured myocytes to determine whether similar changes were observed.

**Figure 7:**
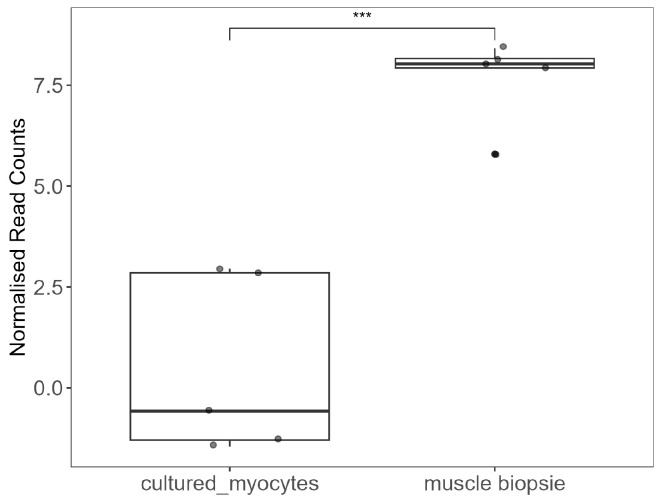
Comparison of *NT5C1A* expression in paired cultured myocytes and biopsied skeletal muscle. Sequencing data was obtained from NCBI GSE17503 (https://www.ncbi.nlm.nih.gov/geo/query/acc.cgi?acc=GSE17503). Pairwise comparison was performed using the Student’s *t* test. Significance level is indicated as follows: *** *p* ≤ 0.001.

A study by Raymond and colleagues compared the transcriptomic signature of cultured myocytes to freshly biopsied skeletal muscle [23]. We analysed their Illumina microarray sequencing dataset to compare *NT5C1A* expression in biopsies and the same samples following a period of culture. In agreement with our present findings, *NT5C1A* was significantly downregulated following time in culture (Figure 7).

**Figure 8:**
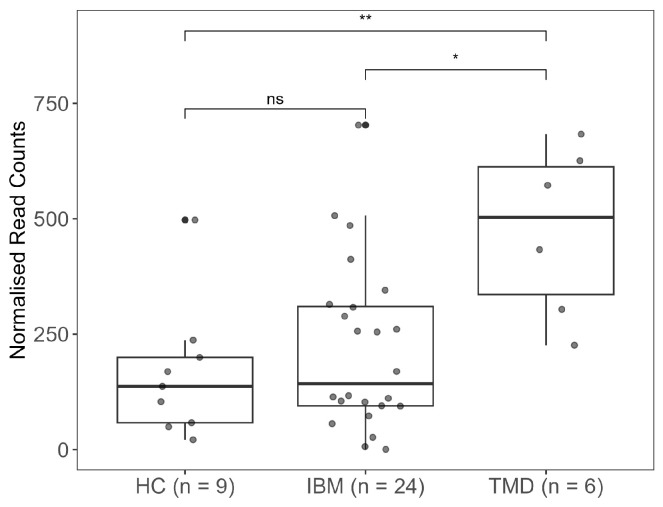
Comparison of *NT5C1A* expression in biopsied skeletal muscle obtained from healthy donors (HC), inclusion body myositis patients (IBM), or tibial muscular dystrophy patients (TMD). Sequencing data were obtained from GSE151757 (https://www.ncbi.nlm.nih.gov/geo/query/acc.cgi?acc=GSE151757). Pairwise comparisons were performed using the Student’s *t* test. Significance levels are indicated as follows: ns, *p* > 0.05; * *p* ≤ 0.05; ** *p* ≤ 0.01.

Moreover, NT5C1A expression appears to vary between individuals. Analysis of the sequencing dataset of Johari and colleagues (GSE151757 (https://www.ncbi.nlm.nih.gov/geo/query/acc.cgi?acc=GSE151757)) [22] revealed substantial variability in *NT5C1A* expression across all sample groups (Figure 8). For example, the mean transcript count in healthy muscle was 164, with a coefficient of variation (CV) of 0.88, while, IBM muscle samples showed similar mean expression of 217 with slightly smaller variability (CV = 0.83). These findings indicate heterogeneous NT5C1A expression in healthy and diseased muscles. Of note, *NT5C1A* expression was increased in dystrophic muscles compared to both healthy and IBM muscles.

## 4. Discussion

This pilot study explored changes in the transcriptome of cultured human myotubes treated with IBM serum containing anti-cN1A antibodies. Based on findings presented in prior reports [14,24], we hypothesised that anti-cN1A antibodies interact with their intracellular enzyme target and induce either its dysfunction or degradation. We reasoned that compensatory mechanisms may increase expression of *NT5C1A* and possibly related genes to counteract the decreased function.

Contrary to our initial hypothesis, we detected no mRNA transcripts of the AMP catabolic enzymes in our model across all treatment conditions. Skeletal muscle is one of the most metabolically active organs, characterised by a high mitochondrial content to sustain the substantial energy demands through adenosine triphosphate (ATP) production. ATP turnover is tightly linked to the maintenance of cellular energy balance, and under conditions of high metabolic activity, ATP is rapidly hydrolysed to ADP and subsequently to AMP. Effective AMP catabolism is critical for preventing its intracellular accumulation and maintenance of the purine nucleotide cycle. In skeletal muscle, AMP catabolism is primarily mediated by two enzymes: AMP deaminase, encoded by *AMPD1*, and cytosolic 5-nucleotidase 1A, encoded by *NT5C1A* [25], and transcripts for both enzymes were severely reduced in the current culture model. In agreement with this observation, Abdelmoez and colleagues reported a significant reduction in *AMPD1* expression in cultured myotubes compared to fresh skeletal muscle tissue ($\log_{2}$ expression = −1.94 vs. 5.85, *p* = $1.9\times 10^{-144}$) [26]. Furthermore, Raymond and co-authors reported a widespread downregulation of metabolic enzymes, including *AMPD1*, in cultured myocytes compared to skeletal muscle tissue [23]. Using this group’s published microarray dataset, we confirmed that *NT5C1A* was also significantly downregulated during the culture process when compared to freshly biopsied muscles. These findings highlight that the in vitro culture environment profoundly alters the transcriptional regulation of key enzymes of the AMP metabolism.

The observations above raise an important question: are the changes in expression of over 1000 genes observed in this study directly related to the presence of anti-cN1A antibodies, which target the enzyme encoded by *NT5C1A*? In this study, we were unable to perform proteomic analyses to confirm the presence of cN1A protein in myotubes. Moreover, the half-life of cN1A protein or its cytoplasmic mRNA has not been reported. Nucleotidases, however, are generally thought to turn over rapidly in response to cellular nucleotide demands. Therefore, without ongoing transcription, it is unlikely that cN1A protein would persist within the myotube cytoplasm for multiple days. Consequently, a direct interaction between cN1A and circulating antibodies is an improbable explanation for the observed gene expression changes. Instead, we speculate that these alterations were driven by soluble factors present in IBM sera that are associated with autoantibody production, such as cytokines, damage-associated molecular patterns, or other inflammatory mediators originating from the muscle microenvironment. Addressing this hypothesis will require further studies comparing the protein composition of sera from anti-cN1A-positive and -negative IBM patients.

Our analysis identified 830 genes that were upregulated by myotube exposure to anti-cN1A-containing serum. More than 10% of these genes encoded metabolic enzymes. Metabolic dysregulation has been previously identified as a pathological hallmark of IBM and a potential source of disease biomarkers [27]. Our observations align with these reports, suggesting that metabolic dysregulation may be even more pronounced in anti-cN1A-positive patients.

Another noteworthy observation was the increased expression of *CTSH* and *CTSZ*, the genes encoding cathepsins H and Z, respectively, following administration of sera containing anti-cN1A antibodies. Cathepsins H (CatH) and Z (CatZ) are members of the cysteine protease family of enzymes that primarily function within lysosomes, where they play essential roles in protein degradation. In the immune system, cathepsins have been suggested to contribute to adaptive immunity by facilitating the processing of complex antigens into peptides, which could then be loaded onto Major Histocompatibility Complex class II (MHCII) molecules for presentation to CD4^+^ T cells [28,29]. They may also influence CD8^+^ T-cell and NK-cell cytotoxicity through activation of perforin and granzymes [30]. Furthermore, cathepsins may modulate innate immune responses by cleaving and activating toll-like receptors [31].

Given the limited number of samples in our study, the observed upregulation of *CTSH* and *CTSZ* could reflect subject-specific effects. We therefore sought to confirm the relevance of this finding to IBM pathology by analysing the RNA sequencing dataset generated by Johari and colleagues, which compared transcriptomes from IBM biopsies with muscles derived from healthy individuals and patients with muscular dystrophy [22]. Importantly, this analysis confirmed a significant increase in *CTSH* and *CTSZ* expression in IBM biopsies relative to control tissues.

The activation of cathepsins is tightly regulated under normal physiological conditions, but dysregulation has been implicated in various pathological states, including cancer, neurodegenerative disorders, and inflammatory diseases (reviewed in [32]). Notably, overexpression of *CTSH* and *CTSZ* have been identified as risk factors for type 1 diabetes, a disease that involves autoreactive T cells targeting pancreatic beta cells [33]. Our study is the first to report a potential link between the upregulation of these genes and IBM. As cathepsins have been suggested to contribute to antigen processing and MHCII presentation, their upregulation could potentially enhance aberrant self-antigen presentation and promote CD4^+^ T cell autoreactivity. CD4^+^ T cells provide help to cognate antigen-specific B cells, facilitating class switching and repertoire maturation, which in turn enables the production of high-affinity antibodies, including self-reactive antibodies. Our findings highlight the need for further investigation into whether increased *CTSH* and *CTSZ* expression is sufficient to promote pathological self-antigen presentation and muscle targeting in IBM. Future studies should aim to elucidate the molecular mechanisms through which these proteases contribute to IBM pathology and to determine whether their overexpression correlates with clinical disease severity.

IBM currently lacks effective therapies and remains largely resistant to conventional immunosuppressive treatments, including corticosteroids, lymphocyte-depleting therapies, IVIG, and cytokine-targeting biologic agents [34]. This resistance may, in part, reflect heterogeneity among patients and underlying pathological processes. Our findings suggest that distinct immunological mechanisms may operate in different patient subsets, with the production of anti-cN1A antibodies possibly representing an outcome of these divergent pathways. These observations underscore the need for robust biomarkers to enable improved patient stratification in clinical trials, allowing therapeutic interventions to be assessed in more biologically homogeneous subgroups. In this context, cathepsins H and Z emerge as promising candidates for evaluation as diagnostic and therapeutic biomarkers in IBM.

We acknowledge a limitation of this pilot study that consisted of a single sequencing dataset within each treatment group. Nevertheless, we endeavoured to strengthen the validity of our findings by including an additional analysis of relevant publicly available sequencing datasets. The downregulation of metabolic enzymes involved in the adenosine salvage pathway, including *NT5C1A* and *AMPD1*, in the 2D myotube culture described here illustrates its limitations as a model of skeletal muscle. Our observations suggest that contractile activity may be required to faithfully recapitulate the metabolic transcriptome of living skeletal muscle. Although animal models remain the most physiologically relevant systems for studying functional skeletal muscle, the principles of ethical research necessitate replacing animal models with alternative approaches whenever possible [35]. In this context, the development of advanced contracting 3D organoid models represents a promising strategy to reproduce more accurately the organisation and function of skeletal muscle tissue [36]. Alternatively, co-cultures of primary human myotubes with murine fibroblast feeder cells subjected to electrical pulse stimulation have been shown to acquire features of normal skeletal muscle function [37]. Such advanced and complex in vitro models may therefore yield gene expression profiles that more closely resemble those of metabolically active skeletal muscle tissue.

## Figures and Tables

**Table 1:** Demographic and clinical characteristics of serum donors.

Diagnosis	α-cN1A Status	IgG Titer ^a^	IgA Titer ^a^	IgM Titer ^a^	Gender	Age (y) ^b^
clinically-defined IBM	Positive	11–25	1–3	0.5–1	Male	79
clinico-pathologically defined IBM	Negative	1.2–3.7	0.1–2.6	0.1–0.7	Male	95
Healthy	NA	NA	NA	NA	Male	67

^a^ Cut-off values: IgG > 4.2, IgA > 5.4, IgM > 2.3. ^b^ at the time of first serum collection.

**Table 2:** Normalised expression of skeletal muscle genes (log(CPM)).

Gene Symbol	Gene Name	HC	IBM^−^	IBM^+^	UT
ACTA1	actin alpha 1, skeletal muscle	5.22	3.62	4.92	7.14
ACTN2	actinin alpha 2	−0.30	4.55	1.22	3.72
MYH1	myosin heavy chain 1	−1.28	−2.03	−3.46	−3.96
MYH2	myosin heavy chain 2	3.06	1.87	−1.37	4.50
MYH7	myosin heavy chain 7	−2.26	2.77	−0.61	−3.96
MYL1	myosin light chain 1	−2.73	5.40	4.15	6.92
MYL2	myosin light chain 2	0.48	4.47	5.05	−3.96
TTN	titin	8.34	7.67	8.34	9.25

Abbreviations: CPM—counts per million, HC—myotubes treated with healthy control serum; IBM^−^—myotubes treated with anti-cN1A-negative IBM serum; IBM^+^—myotubes treated with anti-cN1A-positive IBM serum; UT—myotubes grown in differentiation medium only.

## Data Availability

Data and analysis files can be accessed at https://github.com/myodp/myotube_transcriptome (updated on 26 September 2025).

## Supplementary Materials

The following supporting information can be downloaded at: https://www.mdpi.com/article/10.3390/muscles4040053/s1, Figure S1: Functional classification of the upregulated genes using Protein Analysis THrough Evolutionary Relationships (PANTHER) version 19.0; Table S1: Expression of selected housekeeping genes in logarithmic counts-per-million.
