# Supplementary material for: Altered Transcriptome Signature in Primary Human Myotubes Exposed to Inclusion Body Myositis Serum: A Pilot Case Comparison of Anti-cN1A Positive and Negative Sera
Source: Muscles. 2025 Nov 10;4(4):53. doi: 10.3390/muscles4040053 (PMC12641819; doi:10.3390/muscles4040053)
Supplement: Supplementary file 1 [file muscles-04-00053-s001.zip › muscles-3885903-supplementary.pdf]

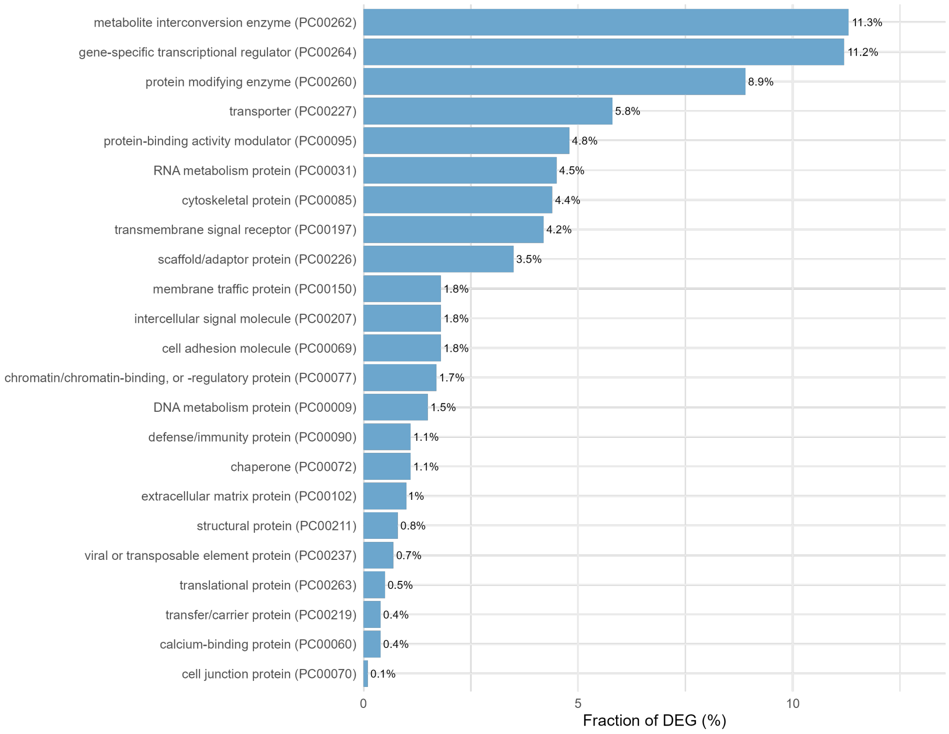

Figure S1: Functional classification of the upregulated genes using Protein Analysis THrough Evolutionary Relationships (PANTHER) version 19.0.

Table S1: Expression of selected housekeeping genes in logarithmic counts-per-million.

**Gene Symbol**	**HC**	**IBM_Neg**	**IBM_Pos**
ACTB	16.46	16.21	16.31
B2M	17.88	18.21	18.05
BLM	6.69	7.03	7.61
GAPDH	13.84	14.06	14.04
PPIA	12.57	11.36	11.65
RPL4	15.49	15.86	15.77
YWHAZ	16.79	16.86	16.87
UBC	14.36	14.21	14.21
EEF1A1	17.12	17.45	17.23
RPL29	12.60	12.64	13.04
RPLP0	13.26	13.74	13.27
PGK1	14.66	14.54	14.48
HMBS	11.19	10.66	9.20
HPRT1	6.02	10.29	10.77
SDHA	12.02	11.87	11.77
TBP	9.18	10.14	10.52
ALAS1	9.77	9.52	9.39
PUM1	9.67	10.51	8.84
GUSB	8.86	9.94	10.54
TFRC	14.51	14.50	14.36
MALAT1	17.93	17.16	17.66
